# Artificial intelligence, human intelligence, and the future of public health

**DOI:** 10.3934/publichealth.2022045

**Published:** 2022-09-16

**Authors:** Sudip Bhattacharya

**Affiliations:** Assistant Professor, Department of Community and Family Medicine, All India Institute of Medical Sciences, Deoghar, Jharkhand, India

**Keywords:** artificial intelligence, emotional intelligence, empathy, sympathy

## Abstract

In this paper, I have described the healthcare problem (maldistribution of doctors) in India. Later, I have introduced the concept of artificial intelligence (AI) and I have described AI technology with various examples, how it is rapidly changing the healthcare scenario across the world. I have also described the various advantages of artificial intelligence technology. At the end of the paper, I have raised some serious concerns regarding complete replacement of human based healthcare technology with artificial intelligence technology. I concluded that there is not the slightest question that AI will influence the future. People must be innovative, insightful, and context-aware for AI to work. This is because humans will continue to contribute value that cannot be reproduced by robots.

## 1. Introduction

Whether healthcare is provided by the government, the private sector or both, the key to improving health outcomes in our nation is the availability of a sufficient number of physicians providing basic healthcare services in disadvantaged regions. In several countries, like India, the situation has changed. Now that there are enough doctors, the problem is incorrect distribution [1].

The new National Health Policy and the National Health Assurance Mission are already in effect. It is time for a comprehensive analysis of the policymakers' viable options for resolving the current dilemma [1].

Approximately 69% of India's population resides in rural areas, where only about 26% of the country's physicians are working, and even then, they are mostly in the private sector. This is too expensive and out of reach for most individuals. In India, almost 833 million people rely on 44,000 physicians [2].

Due to the unequal distribution, an average of 19,000 patients are treated by each physician. This scenario is causing increased out-of-pocket medical expenses. Consequently, 3.3% of the population of India becoming poor on yearly basis [3]. This is solely related to the quantity. Likewise, the issue of care quality is of similar importance. A healthy and sustainable health staff must be accessible 24 hours a day, seven days a week, for best health results.

A study emphasized that the inequitable distribution of doctors in the Indian public sector poses a threat to the health and economic well-being of the people [4].

## 2. Artificial intelligence in non-health sectors

As in other industries, we should explore implementing technology to tackle this issue, such as information technology-enabled self-service kiosks to minimize the bankers' (manual) burden. Beneficially, the aviation and railroad sectors have adopted automation and technology [5].

Now, we inhabit an era of artificial intelligence (AI). AI is, in the most fundamental sense, the natural intelligence of a computer system that is gained via consistent contact with end users (humans). This non-human intelligence can accomplish miracles with an accuracy and reliability of 90 to 96% [5].

In the era of AI-based technology, life has become easier; for instance, Amazon Lab126's Alexa is a household-oriented virtual assistant. It can have features such as voice recognition, voice interaction, music playback, creating to-do lists, providing real-time weather and traffic information, etc. Alexa can also control numerous smart gadgets, like the TV and air conditioner. Alexa's powers can be increased by installing new skills (also known as applications) [6].

## 3. Utilization of artificial intelligence in healthcare

Japan has already employed AI-based robotics to address geriatric care-related commercial challenges (personally programmed). These aid the elderly in doing daily duties, such as taking their prescriptions in the morning and adjusting the temperature of their air conditioner as they rest in bed [7].

It is also useful for detecting glaucoma. Glaucoma, an optic nerve disorder, is considered as one of the primary causes of blindness. Glaucoma is a global public health concern. A steady rise in intraocular pressure leads to pressure atrophy or atrophy, resulting in unilateral or bilateral vision loss. Glaucoma can only be identified by comparing the texture of a normal retinal image to an image of glaucoma. This study employs Haralick characteristics to differentiate between photos of normal and damaged retinas. Glaucoma has been detected with 96% accuracy by utilizing AI (via image extraction and training of the back propagation neural network) features [8],[9].

UE Lifesciences created the iBreastExam (iBE), which is a “portable, clinically proven, non-invasive, painless, and radiation-free instrument” that aids in the early diagnosis of breast lesions at the site of contact. It offers a “map” of breast tissue that detects irregularities that are afterward probed for the presence of carcinomas. Approximately 50,000 women were screened using the iBE over the course of one year; among them, 2251 were found to have breast lumps, 75 of which were malignant. Initially, just a few patients showed up for examinations. But the number climbed dramatically. The (USA) Food and Drug Administration-approved iBreastExam has been used on over 150,000 women in India and in six other countries: Botswana, Indonesia, Mexico, Myanmar, Nepal and Oman. This user-friendly breast cancer screening device can therefore assist underserved/difficult to reach populations and increase the 5-year breast cancer patient survival rates [10].

Currently, robot-assisted surgery is gaining popularity. Surgical robots can perform advanced minimally invasive treatments with better vision, precision and dexterity than laparoscopy [11]. These are successful applications of AI in healthcare. According to the preceding discussion, complex machine learning algorithms, AI and robotics will affect the future of all scientific endeavors.

## 4. Advantages of artificial intelligence

The two categories of human capacity are cognitive and physical. In the past, humans dominated machines because of their cognitive superiority, while robots dominated mostly due to their better physical ability. Thus, most of the manual work in agriculture and industry was automated. As a result, vocations that formerly needed largely cognitive talents, such as learning, analyzing, communicating and understanding human emotions, have developed into “new” occupations [12].

It is essential to understand that the AI revolution is not just about improved technology and supercomputers. Additionally, it is currently propelled by developments in the biological sciences.

The better our understanding of the physiological principles underlying human emotions, desires and decisions, the more competent computers will be when analyzing and predicting human behavior, making them better able to replace human drivers, bankers, and lawyers [13].

Scientists utilized data from neuroscience and behavioral economics to get a deeper grasp of human decision-making a few decades ago. All our choices/decisions/wants (from eating food to wearing clothes) are not the result of strange intuitions, but rather the probability estimated by billions of neurons in a fraction of a second, according to the research findings. Human intuition is nothing more than the recognition of patterns and happenings [14].

Good physicians, surgeons and nurses do not possess supernatural abilities when diagnosing patients. It is like reassessing recurring patterns/events. Typically, they observe the pattern or occurrence learned in medical school and aim to avoid making any mistakes throughout the investigation, diagnosis, therapy or surgery. The study also indicated that the biochemical algorithms of the human brain are not faultless. According to specialists, the molecular algorithms of our neurons are deteriorated, short, outmoded and adaptable [14].

Therefore, even competent surgeons and physicians sometimes make unintentional mistakes. The accompanying explanation demonstrates that AI-enabled doctors can also outperform human doctors in the cognitive domain.

As mentioned above, a physician's ability to identify a problem does not depend on supernatural abilities or intuition. Instead, their brains find biological patterns by evaluating the signs and symptoms of patients. If AI is equipped with the proper sensors and software, it may be able to treat patients with greater precision and dependability than a human physician.

Similarly, if the World Health Organization discovers a new disease or a laboratory creates a novel treatment, it is impractical to tell all traditional physicians about these developments. Compared to this, 10 billion AI-enabled physicians may share and update the same information in a fraction of a second. They may also exchange their thoughts on the new infections or treatments via non-neural networks [15].

A patient in a remote community has access to hundreds of AI-enabled physicians whose relative performance is continuously compared when many algorithms are executed on the same network. The patient may simply seek a second opinion from a “traditional doctor” if he or she is unhappy with the AI-enabled physician's diagnosis [16].

Significant future advantages for human civilization are envisaged. For billions of underprivileged individuals, AI-enabled doctors might provide far improved and more economical care. Due to machine learning and biometric sensors, it may be possible for a poor peasant to receive the same level of healthcare via a smartphone as the wealthiest individual in a modern metropolitan health facility [16]. A practical advantage of AI's non-human capabilities is the ease and speed with which they may be connected and upgraded [15].

## 5. Concerns about artificial intelligence

### 5.1. Concern for job loss

This information technology and biotechnology revolution has the potential to replace human physicians in areas with limited resources [15]. As an example, the present understanding of the basal ganglia, pons, cerebrum and cerebellum by psychiatrists and neurologists may one day allow computers to exceed human psychiatrists and neurologists.

Some may argue that, if we switch to AI-enabled doctors, we may lose the advantages of individual clinical judgment and creativity [16].

A doctor endowed with AI may not only be able to hack humans and outperform them in their uniquely human abilities, but they may also have a competitive advantage over their distinctively non-human skills.

AI's potential is incomprehensible, yet it is also restricted. It seems likely that AI will dominate the professional world on a variety of levels, but the degree of its dominion is questionable.

### 5.2. Ethical concerns

The ethical implications of AI-based healthcare decision-making raise several issues, such as whether there will be sociodemographic biases such racial prejudice and cultural inequalities. How will it impact careers in the healthcare and budgeting fields? Who will be held accountable for any unintended repercussions of AI-driven therapy algorithms or unexpected mistakes made during invasive medical procedures? What might automatic devices in the healthcare industry mean ethically? These inquiries are very important, and it can be difficult to provide ethical responses in the context of AI-enabled healthcare.

Therefore, adopting an accountability framework approach is crucial for the design, development and deployment of AI-based medical technology to better comprehend and address the concerns. This strategy should be centered on promoting human welfare while combining moral standards and cultural ideals. This type of strategy is crucial since AI affects and worries us all. Instead of limiting our analysis to the person, we must consider AI-based medical systems in the context of the complex technological and medical realities and social dynamics.

The following are the proposed strategies to uphold ethics when developing AI-based healthcare systems:

1. When designing AI systems, theological and technological procedures that consider cultural factors should be known as “ethics in design”.

2. Establishment of applied ethics, which focuses more on the behavioral ethics involved in creating and using AI systems in healthcare. Such ethical considerations may center on how humans use and develop AI systems in healthcare, which is likely to influence how AI systems behave digitally in the future.

3. The establishment of ethics for various stakeholders, which might be defined as the norms and regulations that ensure the honesty of all participants as they create and implement AI systems. Understanding these ethical concepts may help us to better understand how sophisticated AI systems might contribute to the population's overall health, which depends on how individual and collective human intelligence behaves in various social circumstances [17],[18].

## 6. Irreplaceable humane roles

We have already covered roles that may be eliminated owing to technical advances, but human labor parts cannot be replicated. Let us focus on something AI cannot achieve. There are certain duties that can only be performed by people.

There are activities requiring creativity, conceptualization and complex strategic planning, as well as the ability to deal with unknown areas and feelings or emotional interactions, that AI is now incapable of performing. Let us now explore skills that are irreplaceable forever.

1. Empathy: Some may argue that animals also exhibit empathy, but they are not replacing humans in the workforce. In contrast to software designed to produce a certain result, people can experience emotions. Personal affinity between a person and an organization is the cornerstone of a professional relationship, despite the seeming contradiction. Humans need a personal relationship that extends beyond the business realm to establish trust and human connection, something that bot technology utterly lacks.

2. Emotional intelligence: Although the AI may be accurate, it lacks intuition and cultural sensitivity, which are human qualities. It will never be able to adapt to the algorithm of human intellect, no matter how exactly it is designed to do a task. For instance, reading a situation or the expression of another person. It lacks the emotional intelligence necessary for individuals to perceive and manage relationships involving emotional communication. A human connection that can read and grasp the issue is always favored over an automated technology that cannot operate or help beyond its programming while providing customer assistance.

3. Creativity is an advantage of being human. AI may boost productivity and efficiency by reducing errors and repetition and replacing manual jobs with intelligent automated alternatives, but it cannot comprehend human psychology. In addition, as the world becomes more AI-enabled, individuals will be able to engage in increasingly innovative activities.

4. Problem-solving outside of a code: Humans may deal with unforeseen uncertainty by studying the issue, e.g., by utilizing critical thinking in challenging situations and creative techniques. Contrary to humans, who can function in a variety of settings and environments, AI-powered devices are limited to their original role. In the distant future, this may be conceivable, but not soon.

## 7. Final words

Finally, we cannot ignore technological advancement. To alleviate human suffering, we must embrace technology with caution. It will be intriguing to watch in the future how much AI-enabled doctors can aid (in diagnosis/treatment/decision-making) in tackling community health concerns and controlling the “maldistribution” of doctors in India. It is time to experiment with AI-enabled physicians in nations with limited resources. There is not the slightest question that AI will influence the future. People must be innovative, insightful and context-aware for AI to work. This is because humans will continue to contribute value that cannot be reproduced by robots. Additionally, it cannot be denied that, no matter how intelligent AI gets, it will never be able to imitate human awareness, thereby reinforcing humans' position at the top of the food chain.

N.B. The opinion expressed in this article is that solely of the author and does not reflect the official position of any other affiliated institution or agency affiliated in the past or present.
